# Professionals’ and Families’ Perspectives on Essential Elements of Shared Decision-Making: A Qualitative Analysis on Families with Multiple and Enduring Problems in Integrated Youth Care

**DOI:** 10.1007/s10488-025-01443-0

**Published:** 2025-04-30

**Authors:** Anne Marie Barnhoorn-Bos, Eva A. Mulder, Laura A. Nooteboom, E. H. Alet Meurs, Robert R.J.M. Vermeiren

**Affiliations:** 1 Department of Child and Adolescent Psychiatry, LUMC Curium, Leiden University Medical Center, Post Box 15, Leiden, 2300 AA The Netherlands; 2 GGZ Rivierduinen Children and Youth, Institute for Mental Health, Postbox 405, Leiden, 2300 AK The Netherlands; 3 Research Institute Child Development and Education, University of Amsterdam, Nieuwe Achtergracht 127, Amsterdam, 1018 WS The Netherlands; 4 Department of child- and adolescent psychiatry & psychosocial care, Meibergdreef 5, AmsterdamUMC, Amsterdam, 1105 AZ The Netherlands; 5 Parnassia Group, Dr. van Welylaan 2, Youz, The Hague, 2566 ER The Netherlands

**Keywords:** Shared Decision-Making, Child and adolescent mental health, Integrated care, Complex problems, Families

## Abstract

Shared decision-making (SDM), known to improve client engagement in care and its quality, is crucial to achieve family-tailored care. However, when providing integrated youth care, SDM can be challenged by the complexity of families’ problems and the multiple family members and professionals involved. Commonly used SDM models may not adequately address these specific challenges. Therefore, this qualitative study explores families’ and professionals’ perspectives on essential elements of SDM with families experiencing multiple and enduring problems on different life domains (e.g., mental health, parenting, financial, and social problems). Semi-structured interviews were conducted with 18 parents, 3 youth, and 22 professionals from Specialist Integrated care Teams in four regions of the Netherlands. A framework method - comprising essential elements of SDM - was applied to systematically code the transcripts both deductively and inductively. Our study shows the nine essential elements of SDM require a specific interpretation in the context of integrated youth care, regarding the diversity of participants in decision-making, the complexity of the problems, and SDM as a continuous process of multiple decisions. In addition, families and professionals mentioned three complementary elements: (1) build collaborative relationships, (2) prioritize problems, goals and actions, and (3) interprofessional consultation. Thus, in integrated youth care professionals are recommended to consider SDM as a cyclical process of larger and smaller decisions and take time to build collaborative relationships with families and the care network. Throughout the care process families and professionals balance their mutual roles in decision-making in line with the changing needs and preferences of families.

**Supplementary Information:**

The online version contains supplementary material available at 10.1007/s10488-025-01443-0.

## Introduction

Families experiencing multiple and enduring problems (such as a combination of mental health, parenting, financial, and social problems of both youth and parents) frequently receive care from various care providers with a focus on issues in different life domains (Bodden & Deković, 2016; Holwerda et al., 2014). However, due to a lack of coherence among care services and insufficient involvement of families, the support provided often fails to match families’ needs and preferences (Nooteboom et al., 2021; Rijksoverheid, 2018; Tausendfreund et al., 2016). Shared decision-making (SDM) can ensure engagement of all stakeholders (e.g., family members and professionals) in decision-making, leading to family-tailored care (Fitzpatrick et al., 2022; Menear et al., 2022; Pii et al., 2020). However, commonly used SDM models may not adequately address the specific challenges of SDM with families with multiple and enduring problems. Therefore, we explored families’ and professionals’ perspectives on essential elements of SDM with families with multiple and enduring problems.

In SDM, professionals and families make decisions on care in partnership, incorporating evidence-based practices, clinical perspectives, and the families’ preferences and values (Bjønness et al., 2020). Decisions involve both the process and content of care (Liverpool et al., 2021). SDM’s underlying principles of autonomy, empowerment, and value-based care, are well aligned with families’ needs for personalized, tailored care, and active involvement in the care process (Box et al., 2023; Sheikh et al., 2024; Zhang et al., 2019). Moreover, SDM is known to improve client engagement in care and it’s quality (Butler et al., 2015; Langer et al., 2022; Liverpool et al., 2021; Simmons et al., 2011). Several SDM models are developed to support decision-making of clients and professionals (Bomhof-Roordink et al., 2019; Hayes et al., 2019; Stiggelbout et al., 2015). Most commonly used models are informed by SDM models of Charles et al. (1997), Elwyn et al. (2017), and Makoul and Clayman (2006), whose model entails nine essential elements as necessary components to ensure SDM is taking place: (1) Define or explain problem, (2) Present options, (3) Discuss pros and cons, (4) Assess clients’ values or preferences, (5) Discuss client ability or self-efficacy to follow through with a plan, (6) Provide professional knowledge or recommendations, (7) Check or clarify understanding of facts and perspectives, (8) Make or explicitly defer decision for a later time, (9) Arrange follow-up to evaluate the effectiveness of decisions, make deferred decisions or revise the treatment plan (Bomhof-Roordink et al., 2019; Makoul & Clayman, 2006). These elements seem robust, as other developed models include the same elements and also more recent systematic reviews of SDM models, including in adult mental health care, yield largely similar essential components of SDM (Bomhof-Roordink et al., 2019; Zisman-Ilani et al., 2017). However, since most models are initially developed for adult (mental) healthcare, they may not adequately support SDM with families with multiple and enduring problems (Bomhof-Roordink et al., 2019). Indeed, making shared decisions with families experiencing multiple and enduring problems presents professionals with specific challenges related to: (1) the context of youth and their families, (2) the complexity of the problems, and (3) the integrated care context in which multiple professionals are involved (Hayes et al., 2020; Liverpool et al., 2021; Cheng et al., 2017

First, SDM in the *context of youth and their families* involves more participants than only the youth in care, including parents, stepparents, siblings, and other family members, as well as other important people from the social network. This implies multiple opinions and preferences to incorporate in the decision-making process (Fitzpatrick et al., 2022; Liverpool et al., 2021). In particular, relational problems and conflicting opinions between parents and youth, or among parents, complicate decision-making (Hayes et al., 2020; Nooteboom et al., 2020; Simmons et al., 2011). Moreover, professionals need to consider youths’ maturity in making choices, even more when they face intellectual impairments due to mental or cognitive problems (Cheng et al., 2017; Hayes et al., 2019).

Second, SDM with families with multiple and enduring problems is impacted by the *complexity of problems.* Specifically, the interrelatedness of problems in these families complicates a clear assessment of underlying causes and treatment options (Raad voor Volksgezondheid en Samenleving, 2020; Tausendfreund et al., 2016). Furthermore, the endurance and variability of problems generates a long-term care process with both regular and crisis-driven moments of choice (Poitras et al., 2020). As a result, prioritizing goals and actions is more challenging, impeding the process of collaboratively determining the focus of support (Nooteboom et al., 2021).

Last, since the multiple needs of families often exceed the expertise and possibilities of a single professional or care service, an *integrated care context* of different professionals and care services (e.g., in mental health, youth and parenting support, and intellectual disabilities) providing care on various life domains of families, influences the SDM process (Nooteboom et al., 2020; Pannebakker et al., 2018). To provide integrated care, essential to families with multiple and enduring problems, professionals aim for coherent, coordinated and continuous support, across different care levels and services and tailored to the needs of families (Nooteboom et al., 2021). Interprofessional shared decision-making - key to integrated care - can be complicated by differences in values, visions, and interests of both professionals and their organizations (Cloin et al., 2022; Kebe et al., 2020; Stacey et al., 2014). Especially in high-risk situations– such as suspected maltreatment or neglect - professionals relatively often disagree about the assessment of families’ situation and about the decisions to be made (Bartelink et al., 2018). These conflicting perspectives on high-risk situations hinder professionals in collaboratively assessing risks and execute safety measures (Berge et al., 2012; Knutsson & Schön, 2020).

Given the combination of these challenges which is specific to families with multiple and enduring problems, commonly used models (including Makoul and Clayman’s) may not adequately address the specific needs of these families in SDM. Therefore, in this qualitative study we aim to explore the perspectives of families and professionals on essential elements of SDM in the context of integrated youth care. With this knowledge, professionals can potentially complement the known essential elements of SDM with specific needs and preferences of families in integrated youth care.

## Method

### Setting

This study was part of the qualitative research project ‘The specialist nearby?!’ (2020–2022) in which we collected data in five Specialist Integrated care Teams (SITs) in four regions (i.e., The Hague, Midden Holland, Alphen aan den Rijn and Katwijk) of the Netherlands. In these fully integrated care teams, professionals from various youth and adult specialized services (e.g. in the domains of mental health, youth and parenting support, intellectual disabilities, and addiction) collaborate on a daily basis to provide outpatient support to families with multiple and enduring problems. Families are referred to SITs from other primary or specialized care services, often after a long history of care. The teams include child and parent social workers, psychologists, and psychiatrists. SITs provide diagnostics, treatment, and counseling for youth (4–18 years old, e.g. for mental health, social, and school problems) and their parents (e.g., for parenting, social, and financial problems) and work locally to deliver specialized care close to families. Their working method can be characterized by a broad view on family functioning on different life domains, an integration of specialist perspectives in support of families, and flexible and timely delivery of specialized care based on the preferences and needs of families. The Medical Ethics Review Board of Leiden University Medical Center concluded the research project, including this sub study, was not subject to the Medical Research Involving Human Subject Act (WMO) and complied with the Netherlands Code of Conduct for Research Integrity (N20.200). The Consolidated criteria for Reporting Qualitative Research (COREQ) were applied to promote transparency and ensure clear and comprehensive reporting of the study methods (Tong et al., 2007).

In this study, a qualitative design comprising semi-structured interviews was applied, enabling us to gather perspectives and experiences on SDM in a setting not previously studied (Ritchie et al., 2013). By the triangulation of perspectives of participants (i.e., parents, youth, and professionals), the credibility of our findings was fostered (Daniel, 2019; Onwuegbuzie & Leech, 2007).

### Participants

Participants were recruited among parents and youth receiving care from SITs and professionals working in SITs. To obtain a broad range of perspectives, we applied a purposive heterogeneous sampling method in which we predetermined the number of participants in each group (parents and youth were counted as one group, i.e., the families’ perspective) and aimed on including participants with a variety of demographic characteristics and backgrounds (Etikan et al., 2016; Robinson, 2014). Concerning families, we aimed for a representative mix of both parents and youth, with variation in gender, cultural background, family status, and educational level. Within the group of professionals, we included participants with varying expertise, occupation, educational level, work experience, and gender as much as possible. To reach the families, who are often highly burdened by their problems, recruitment of parents and youth was conducted directly by professionals from the teams. Overall, professionals felt inhibited to invite families by their fear of overburdening parents or youth or doubts about parents or youths’ ability to participate. In achieving the required number of families, eventually we asked professionals to provide all families receiving care from the SITs with an invitation to participate in an interview by means of an accessible flyer, formulated in collaboration with a parent representative. The group of parents and youth willing to participate, largely matched the variation in demographics and background we aimed for. However, the number of youth participating is limited, for which we lack a clear explanation. Possibly, youths did not feel capable or were not motivated to participate in research. Professionals were invited (by email, telephone or face-to-face) by the researchers (EH, AB) or the contact persons of the SITs. Families and professionals were provided with verbal information and an additional letter describing the project and process of interviewing (audio-taping, confidentiality, and the right to withdraw at any time). After their written informed consent, participants were contacted to schedule the interview at their preferred location (at home, the team’s office or the researchers’ office) and modality (online or face-to-face). Necessary demographic data of all participants were documented before or during the interview, such as gender, age, cultural background, and educational level. Parents and youth received a gift voucher (of 20 euro) in acknowledgement of their participation.

### Data Collection

Data were collected between March 2021 and April 2022 through semi-structured interviews by two researchers (EH and AB). On the basis of previous studies on SDM in integrated youth care (Bjønness et al., 2020; Elwyn et al., 2017; Langer & Jensen-Doss, 2018; Nooteboom et al., 2020), a topic list with open-ended questions was formulated (see appendix A). The topic list included open questions (not referring to a specific SDM model or limited to the setting of SITs) on how professionals and families reach shared decisions, which elements they consider important in this process, and roles of families and professionals in SDM. With a parent representative (CT) the topic list was modified to fit the language and experiences of parents and youth. Interviews (mean duration of one hour) were conducted by two researchers (EH and AB), audio-recorded, and afterwards pseudonymized and transcribed (verbatim) by the researchers (AB and EH) and students (two of Medical University and one of University of Applied Sciences). As the interviews were conducted in Dutch, the presented quotes have been translated from Dutch to English by the researcher (AB).

### Analysis

All transcripts were imported into ATLAS.ti (version 9), a qualitative data analysis software for labeling and organizing textual data. A theory-driven framework method was applied to systematically code the transcripts both deductively and inductively (Gale et al., 2013; Smith & Firth, 2011). The coding framework was initially formed by Makoul and Clayman’s nine essential elements of SDM (see Appendix A) (Makoul & Clayman, 2006), and supplemented with open codes of other important elements in SDM described by families and professionals. In an iterative process throughout the data analysis, the coding framework was discussed and refined by the researchers (AB and LN), adjusting code descriptions or adding new codes (Gale et al., 2013; Smith & Firth, 2011). All interviews were coded, and after coding 25 interviews (an equal number of both families and professionals), no new themes emerged, indicating thematic saturation (Varpio et al., 2017). However, depth and nuances within the themes emerged when coding the remaining interviews. In summarizing and synthesizing the coded data, we either assigned open codes to the existing nine essential elements or formed complementary elements from these codes. Each element was further analyzed by describing patterns and themes, regarding its practical interpretation in integrated youth care, roles of professionals and families in decision-making, and potential differences in perspectives of parents, youth, and professionals (Smith & Firth, 2011).

Additionally, a summative content analysis of both the existing and complementary elements was performed to identify patterns regarding which elements are mentioned by the different groups of participants: parents, youth, and professionals (Elo & Kyngäs, 2008; Hsieh & Shannon, 2005).

A reflexive stance considering the researchers’ perspectives in generating and interpreting the data was fostered by keeping a research journal, noting reflections after each interview and observation, and by reflective meetings with the researchers (AB, LN, EH) and a parent representative (CT) (not a study-participant) (Olmos-Vega et al., 2022). During these meetings the researchers reflected on their roles, interests and biases from different backgrounds and expertise (AB also working clinically in youth mental health care, LN as expert on integrated care, EH conducting doctoral research on integrated care, and CT contributing from a parent experiential perspective). Informant feedback to increase internal credibility was obtained through four learnings sessions on preliminary results with the participating professionals, managers, and policymakers, including one session with parents and youth representatives (Onwuegbuzie & Leech, 2007). These sessions also served as a member check (Varpio et al., 2017). Our reflexivity throughout the study with families and professionals guided us in interpreting the data in the light of families’ and professionals’ daily reality, which allowed for more nuances and depth.

## Results

This section starts with a short description of demographics of participants. Subsequently, we outline the qualitative findings of our framework analysis, describing the nine essential and the complementary elements of SDM in the context of integrated youth care. Finally, we present the qualitative results of our summative content analysis.

### Participant Demographics

In total, 43 interviews were conducted with parents (*N* = 18), youth (*N* = 3), and professionals (*N* = 22). Four parent couples wished to be interviewed together and two parents requested to be interviewed in the presence of a professional for support. Demographics of the parents, youth, and professionals can be found in Appendix B.

The participating youth were all adolescent-aged yet varied in gender (identified male (66,7%) and female (33,3%)), educational level, and family structure. Parents varied in age (30–39 (11,1%), 40–49 (44,5%), 50–59 (33,3%) years old, unknown (11,1%)), educational level, and family structure (e.g. two-parent (66,7%) and single parent (33,3%) households, raising one (11,1%), two (50,1%), or three and more (38,8%) children). Their cultural background was mainly Western (94,5%) and the majority of participating parents were identified female (77,8%). The group of participating professionals showed variation in age (30–39 (36%), 40–49 (36%), 50–59 (23%), and 60–69 (5%) years old), work experience, educational level, expertise (e.g. youth mental health (23%), youth and parenting support (45%), intellectual disabilities care (23%), and youth health service (9%)), and occupation (i.e. child and parent social workers (59%), psychologists (22%), systemic therapist (5%), pediatric nurse (5%), and child psychiatrist and youth physician (9%)). The majority of professionals were identified female (95%).

### Framework Analysis

In our framework analysis, the elements parents, youth, and professionals described as important predominantly fit within Makoul and Clayman’s SDM model of nine essential elements. However, these elements require a specific interpretation in the context of integrated youth care, regarding the diversity of participants in decision-making, the complexity of the problems, and SDM as a continuous process of multiple decisions. In addition, parents, youth, and professionals mentioned three elements complementary to the original model: (1) build collaborative relationships, (2) prioritize problems, goals and actions, and (3) interprofessional consultation.

In the following section, the nine existing and three complementary elements of SDM in the context of integrated youth care are presented. First, Table 1 summarizes the specific interpretation of Makoul and Clayman’s nine essential elements in integrated youth care. Then, the three complementary elements are presented in Table 2. Finally, based on our framework analysis, for each element the key points of its implementation in the context of integrated youth care, according to parents, youth, and professionals, are described. We highlight roles of professionals and families in decision-making and differences in perspectives between parents, youth or professionals, if mentioned by participants. A perspective referred to as a families’ implies that it was described by both parents and youth.

**Table 1:** Interpretation of Makoul & Claymans (2006) essential elements of SDM in the context of integrated youth care

Essential Elements of SDM according to Makoul & Clayman ^a^	Essential Elements of SDM in the Context of Integrated Youth Care	Summative Content Analysis	
Define or explain problem	Clarify multiple problems of the different family members and on various life domains. Also define the family’s goals and what is needed to achieve them. Explore the context and interrelatedness of the multiple problems through diagnostic assessment.	Professionals	*n* = 16
		Parents	*n* = 18
		Youth	*n* = 3
		Total	*n* = 37
Present options	Present options of care and brainstorm on solutions to the problems with the family. In addition to formal care options, consider which support can be provided by others, for example the informal network.	Professionals	*n* = 12
		Parents	*n* = 15
		Youth	*n* = 3
		Total	*n* = 30
Discuss pros and cons (benefits/risks/costs)	Discuss pros and cons of the options in terms of benefits and costs for the whole family, e.g., regarding the required effort and time.	Professionals	*n* = 2
		Parents	*n* = 7
		Youth	*n* = 0
		Total	*n* = 9
Assess clients’ values or preferences	Assess preferences of the various family members regarding the type and form of support (e.g., place, intensity, frequency) and the collaborative relationship between family and professionals. Take into account their experiential knowledge.	Professionals	*n* = 17
		Parents	*n* = 18
		Youth	*n* = 3
		Total	*n* = 38
Discuss client ability or self-efficacy to follow through with a plan	Consider whether a care plan is feasible for the whole family, regarding the required effort in time and focus, the achievability of the intended behavioral change. Note the required skills and motivation of a youth or parent to participate in a specific treatment.	Professionals	*n* = 6
		Parents	*n* = 9
		Youth	*n* = 0
		Total	*n* = 15
Provide professional knowledge or recommendations	Provide professional knowledge and advice on the problems and the care plan. Share professional reflections and concerns on the interaction of problems, the family dynamics, and the collaborative relationship.	Professionals	*n* = 13
		Parents	*n* = 18
		Youth	*n* = 3
		Total	*n* = 34
Check or clarify understanding of facts and perspectives	Throughout the care process verify the family’s understanding of the problems and goals of care. Provide additional explanation or recapitulation, particularly to family members with intellectual disabilities.	Professionals	*n* = 1
		Parents	*n* = 6
		Youth	*n* = 0
		Total	*n* = 7
Make or explicitly defer decision for a later time	Make a joint decision on the support to be provided and its implementation.	Professionals	*n* = 14
		Parents	*n* = 8
		Youth	*n* = 2
		Total	*n* = 24
Arrange follow-up to evaluate the effectiveness of decisions, make deferred decisions or revise the treatment plan.	Continuously evaluate and adjust the care plan through evaluation meetings. Ensure that goals and support remain in line with the changing needs and preferences of the family over time. Evaluate both formally at set times, and informally throughout the support.	Professionals	*n* = 11
		Parents	*n* = 12
		Youth	*n* = 1
		Total	*n* = 24

*n* = number of participants quoting this element. Total number of participants *N* = 43, professionals *N* = 22, parents *N* = 18, youth *N* = 3

^a^ Makoul & Clayman (2006)

**Table 2:** Complementary elements of SDM in the context of integrated youth care

Complementary Element	Description of the Element	Summative Content Analysis	
Build collaborative relationships	Build collaborative relationships between the various family members and professionals. Learn to know the family, their needs and preferences and take time to make a connection.	Professionals	*n* = 17
		Parents	*n* = 18
		Youth	*n* = 3
		Total	*n* = 38
Prioritize problems, goals and actions	Prioritize the order in which problems and goals should be addressed. Assign actions and responsibilities to the different professionals and family members.	Professionals	*n* = 7
		Parents	*n* = 7
		Youth	*n* = 0
		Total	*n* = 14
Interprofessional consultation	Consult other professionals on problems, goals, and options of support of the family, for example in multidisciplinary meetings. Strive for joint decisions on the support to be provided and its implementation.	Professionals	*n* = 12
		Parents	*n* = 2
		Youth	*n* = 0
		Total	*n* = 14

*n* = number of participants quoting this element. Total number of participants *N* = 43, professionals *N* = 22, parents *N* = 18, youth *N* = 3

#### Nine Essential Elements

***1 Define or Explain Problem.*** To align to the multitude and complexity of families’ problems, participants described four key points regarding this element. First, in defining problems and determining goals a broad view is essential according to families and professionals. What do different family members (i.e. youth in care, parents, siblings) need in various life domains, such as mental health, family relations, education or work, household and finances? This implies both the short term (e.g., urgent needs and crisis intervention) and the long term (e.g. changing family patterns and increasing self-reliance). Second, problem definition and goal setting was described as a process: family and professionals figure out what the family wants and needs as they talk and work together. Third, due to the complexity of the problems, throughout the care process new problems or needs arise that require decisions to be made. As a result, professionals and families regularly return to this element to redefine problems and goals. Finally, families and professionals alike reported professionals have an active role in identifying needs and increasing families’ awareness of significant problems and goals. However, the goals determined must be families’ own and not enforced by professionals.

“In any case, you have to start with the goals of which families themselves say: this is what bothers us. And along the way, maybe there will be room for the goals we see.” Professional 1.

***2 Present Options.*** In the context of families facing multiple and enduring problems, presenting options of care or other services to implement the determined goals was described by four key points. First, options of care are suggested for different family members and in various life domains, with a broad view on different types of care (both inside and outside SITs), social services or informal support. The options should take into account both the various family members and life domains, as well as the time dimension: options of care for both the short and long term. Second, because of the complexity of the problems, families viewed professionals as primarily skilled in and responsible for considering care options. However, families mentioned professionals should be receptive to ideas from the family and consider them seriously. Third, a number of professionals noted they first discuss and pre-select options with other professionals before presenting (a selection of) options to the family. Finally, due to the complexity of the problems and waiting lists in (youth) services, professionals mentioned that limited availability of options must be taken into account.

“My needs she translates, she can identify what I am missing and with her knowledge find it for me.” Parent 4.

***3 Discuss Pros and Cons (benefits/risks/costs).*** Discussing pros and cons of the presented care options with families was scarcely mentioned by participants. Some parents described discussing pros and cons of options in terms of feasibility of the care plan, considering families’ current capabilities and strengths. Few professionals reported discussing pros and cons of care options. If they did, they discussed them mainly with other professionals instead of with families.

“Everything is discussed. They do indicate clearly: from our point of view this seems like the better choice or this may seem wiser. But maybe this might appeal more to you.” Parent 5.

***4 Assess Families’ Values or Preferences.*** Because of the long-term nature of problems, families built up considerable experience with care, what needs to be considered in this element. First, parents and professionals emphasized the value of including parental experiential knowledge about their child, family dynamics and previous support in decision-making. Second, professionals as well as families reported family members may be unaware of their needs and values or may not express them adequately. This requires professionals to identify and describe families’ unspoken needs for them. Finally, professionals valued the belief jointly exploring families’ values and preferences is key in finding support that suits families. Even more important, families highlighted the importance of professionals expressing and embodying this belief.

“That really serious listening is done, what you as a parent think it has to do with, or what makes things the way they are. And also what works and what won’t work.” Parent 13.

***5 Discuss Client Ability or Self-efficacy to Follow Through with a Plan.*** Instead of focusing on the ability of one individual client, a broad view is essential in this element. First, professionals and parents described this broad view in assessing the ability of different family members as well as the feasibility for the family as a whole. Second, parents emphasized the importance of verifying that advice given to youth is also manageable for the parents. After all, parents are responsible for their child. Moreover, parents may be needed in the implementation of a youth’s care plan or experience the consequences of advice given to youth in their family life.

“It would be nice if you ask all three parties, child, parent and professional: is it feasible for you, what do you think? It is easily forgotten that parents are the ones who have to implement it.” Parent 6.

***6 Provide Professional Knowledge or Recommendations.*** Because of the complexity of families’ problems and of the care system involved, families and professionals emphasized the importance of providing professional knowledge in SDM, though four key points. First, professionals contribute essential expertise in defining and clarifying the interaction of problems. Second, their professional knowledge and experience is needed in suggesting options of support for families’ problems. Third, parents mentioned professionals provide advice on care options in both the short term and the long term. In the short term parents gain concrete advice on current problems. In the long term, parents benefit from professional’s helicopter view of possible barriers and necessary support in new stages of life. Fourth, both parents and youth emphasized professionals should not enforce their own views in the decision-making process. Thus, a balance must be found between professional knowledge on the one hand and families’ values, preferences and experiential knowledge on the other.

“I like that they let me talk out first and take my view, but give feedback and show a second side. I can sometimes think pretty black and white.” Youth 1.

***7 Check or Clarify Understanding of Facts and Perspectives.*** Both professionals and parents mentioned the importance of checking understanding of facts and perspectives through the decision-making process. This element is of specific concern, considering the prevalent intellectual disabilities among both parents and youth from families with multiple and enduring problems. Consequently, parents or youth may require more frequent recapitulation or additional explanation of problems, goals, and options of care. Furthermore, both professionals and parents noted that limited motivation for support may also be related to insufficient understanding. Therefore, they described motivational issues as a cue for professionals to check families’ understanding.

“A youth needs to understand it, because that’s the way they know whether they can go along with it or not. I also need to understand what you (professional) want to do. If I think, ‘Okay, that’s what you want to achieve and that seems beneficial to me’, then that’s fine by me.” Parent 6.

***8 Make or Explicitly Defer Decision to a Later Time.*** In the integrated context of care for families with multiple problems, making a decision involves various stakeholders (e.g., family members and professionals from different organizations). These stakeholders can hold varying opinions, and have different authorities and roles. We identified three different forms of decision-making, mentioned by participants, in which professionals and families have differing roles. In the first form, professionals make a decision in interprofessional consultation, before presenting it to the family for approval. In the second form of decision-making, professionals and family jointly reach a decision in consultation. In the third form professionals decide for the family, to protect or compensate parents or youth in case of safety issues. Moreover, due to the multitude of problems and decisions to make, both parents and professionals recommended to prioritize which decisions are made with and without the family. In this way, overwhelming families with information and consultations is prevented.

“It’s just both. I get to decide what I want, but then again I don’t. Because he (professional) decides about it with me. Because if I get to decide 100% what I want to do myself, then what is he really there for?” Youth 2.

***9 Arrange Follow-up to Evaluate the Effectiveness of Decisions***,*** Make Deferred Decisions or Revise the Treatment Plan.*** In the multitude and variability of problems, regular evaluations may bring structure to the decision-making process and support professionals adhering to goals. According to professionals, evaluations initiate a cycle of decision-making, trying out, evaluating, and adjusting the care plan. Moreover, both parents and professionals emphasized joint evaluations can enhance families’ involvement and motivation in care.

“We also have regular evaluations to look back and see what we will focus on in the coming weeks. In that way, the approach is also very flexible and we can adjust things as needed at that moment.” Parent 1.

#### Three Complementary Elements

In addition to the nine essential elements, we found three complementary elements families and professionals considered valuable in SDM.

***1 Build Collaborative Relationships.*** A strong focus on building collaborative relationships is needed since many families have negative and often disappointing experiences with support. Learning to know families, their needs and preferences, making a connection, and building trust were all described as needed to eventually make decisions together. Moreover, families and professionals mentioned that making shared decisions from partnership also contributes to building mutual trust.

*“*I always start by connecting with parents. That parents understand why you are there, what you are doing, what your approach is, by being understanding, apologizing, all those kinds of things.” Professional 18.

***2 Prioritize Problems***,*** Goals and Actions.*** In the multitude of problems and goals, prioritization was described as a complementary element for SDM. As first key point in this element, professionals and parents reported prioritization is often based on professionals’ expertise on logical sequences in addressing problems. For example, by tackling financial problems first to create room for counseling or treatment. Second, priority can also be given to which problem weighs most heavily on families or to which intervention families are most motivated for. Third, parents and professionals emphasized families need professionals’ support to gain overview on the various problems and, actually, be able to prioritize.

“And you try to figure out with the youth: what needs to be done and what to do first… Yes, sometimes a youth has to go to school because we have compulsory education. But if a youth has so much trauma, for example, you can put a youth in class very nicely but it’s no use. Then something else will have to happen first.” Professional 10.

***3 Interprofessional Consultation***. Since there are often various professionals involved, interprofessional consultation takes place in various stages of decision-making and parallel to the decision-making process with families. This element was mainly mentioned by professionals. As first key point, they described to benefit from different perspectives and expertise when dealing with complex problems in families and making delicate, challenging decisions. Second, professionals reported mutual coordination between colleagues and with other care providers ensures an integrated care plan matching families’ needs. Third, professionals described different perspectives on interprofessional consultation. Some professionals deliberately pre-discuss decisions with other professionals to avoid unnecessary burden on families and present families a sorted-out care plan. They inform families in advance and afterwards of the issues discussed. For other professionals, interprofessional consultation in absence of families seemed to be a standard and unconscious practice in decision-making, after which the decision is presented to families for approval.

“We often start looking first to see if the care professionals agree with the plan and only then present it to the family. Because otherwise you end up with a kind of loose plan that only makes these families even more unstable. Because they are already so vulnerable.” Professional 8.

#### Summative Content Analysis

In a summative content analysis (see Tables 1 and 2), we found patterns regarding the numbers of participants of each group quoting the element concerned. Overall, most elements were mentioned by parents, youth, and professionals. However, there were differences between the elements in the number of participants who quoted the element. For example, the elements *Define or explain problem*, *Present options,* and *Assess clients values and preferences* were quoted by considerably more participants than the elements *Discuss pros and cons* and *Check or clarify understanding*. Moreover, there were differences between the groups in some elements. To illustrate, the complementary element *Interprofessional consultation* was quoted considerably more often by professionals than by parents and youth. However, we found participants within a group hold varying preferences regarding the implementation of the element, as described in the framework analysis. In addition, we found parents differed in the importance they attribute to an element. For example, one parent emphasized regular follow-ups as crucial in SDM, while another parent valued the inclusion of families’ experiential knowledge during SDM.

## Discussion

In this qualitative study we explored families’ and professionals’ perspectives on essential elements of shared decision-making (SDM) with families with multiple and enduring problems. The elements of SDM described by parents, youth, and professionals as important in SDM predominantly fit within Makoul and Clayman’s SDM model of nine essential elements. However, these elements require a specific interpretation in the context of integrated youth care, regarding the diversity of participants in decision-making, the complexity of the problems, and SDM as a continuous process of multiple decisions. In addition, both families and professionals mentioned three elements complementary to the original model: (1) build collaborative relationships, (2) prioritize problems, goals and actions, and (3) interprofessional consultation. Adhering to Makoul and Clayman’s model, all elements should be considered as essential components to ensure that SDM takes place, but not necessarily in a fixed sequence or exclusively at one point in the SDM process. Moreover, according to our summative content analysis, families differ in the value they attribute to an element. Therefore, a tailored approach is needed, in which professionals align the implementation of the elements with the particular family.

Thus, as expected, the integrated youth care context of families with multiple and enduring problems requires a specific approach in SDM. In this discussion, we address the key points from this study, describe how our findings align with or distinct from previous research, and outline the implications for practice related to the specific context of families with multiple and enduring problems, regarding (1) the context of youth and their families, (2) the complexity and endurance of the problems, and (3) the integrated care context in which multiple professionals are involved. Several recommendations may also be relevant in other settings, such as youth (mental) health care (e.g., involving parents and other family members in SDM), adult mental health care (e.g., SDM in complex and enduring psychiatric problems) or health care (e.g., SDM in chronic illness or multiple needs in medical, mental, and social domains). However, as SDM with families experiencing complex problems is hampered by a combination of challenges, in integrated youth care it seems important to take all three described areas into account.

### Practical Implications

**SDM with Youth and their Families.** To take into account the diversity of wishes and needs of multiple family members on various life domains, professionals are recommended to consider a broad view on family members and life domains throughout the SDM process. Not only in defining problems and goals, but also in presenting options of care, assessing values and preferences, and, eventually, making a joint decision. However, the care plan should transcend the needs of different family members, and also address the feasibility of the plan for the whole family.

Moreover, building collaborative relationships appears crucial in decision-making with families with multiple and enduring problems. Due to families’ previous, often adverse experiences with support, building collaborative relationships proves conditional to making decisions together. In addition, the collaborative relationship appears to be strengthened by the decision-making process. For example by reflecting with families on their issues and goals, exploring their values, preferences, and experiential knowledge, and by making decisions together. Previous studies also suggest the positive impact of SDM on the collaborative relationship and alliance between client and professional (Bunn et al., 2018; Vooijs et al., 2021). Our study specifically highlights the importance of professionals’ intrinsic belief only through partnership with families the provided support can be successful.

**SDM in the Complexity and Endurance of Problems.** Because of the complexity, attention should be given to the explanation of the problems to families. This requires not only defining the multiple problems, but also clarifying the contributing and maintaining factors. This outcome supports recommendations from previous research in youth care to create a shared explanatory analysis and make choices in support accordingly (Spijk-de Jonge et al., 2022; Tempel et al., 2022).

Second, considering the long-term nature of the problems, a focus on both the short and long term is key in decision-making. Short-term decisions ensure the necessary actions in crisis situations and fulfil families’ needs for prompt support. A long-term (helicopter) view proves beneficial to analyze and change dysfunctional patterns in families and their care system, in order to create sustainable solutions for the family.

Third, due to the multitude and endurance of problems, SDM appears not to be limited to the start of the care process but to entail a continuous and cyclical process of multiple larger and smaller decisions to be made with the family (Verwijmeren & Grootens, 2023). We found regular evaluations, both formal and informal, can give structure to this complex process and keep families connected to their own goals and needs. In addition, prioritization of problems, goals and actions proves key to maintain overview in the multitude of problems and to determine the focus of support. Therefore, in line with previous research, we recommend to regularly review with families whether the care plan still fits their current problems, needs and priorities (Nooteboom et al., 2021). Beyond planning formal evaluations with families, informal evaluation during counseling sessions with families may be a constant mindset of professionals throughout the support. In this way, professionals and families ensure that the support provided matches families’ current needs, while leaving room for newly arising problems needing decision-making. This outcome is in line with the movement towards feedback-informed treatment. A feedback- informed approach seeks to create continuous feedback loops between clients and service providers. In this approach, professionals use client’s in-session feedback to evaluate, inform, and tailor the provided support in order to maximize client-therapist and client-therapy fit (Tam & Ronan, 2017).

**SDM in an Integrated Context with Multiple Professionals**. As several professionals with diverse backgrounds are involved with families, interprofessional consultation is used in SDM to integrate different professional perspectives into an appropriate family care plan. However, in many cases, this implies professional-made care plans are presented to families for approval. Several studies on SDM likewise found professionals describing their way of working as “already doing SDM”, whereas their practice could be considered rather as client consultation in order to find agreement to professionals’ advice (Farrelly et al., 2016; Hayes et al., 2019; Knutsson & Schön, 2020). As a result, clients frequently experience a lack of involvement in the decision-making process (Farrelly et al., 2016; Ten Brummelaar et al., 2018). In our study, however, several parents mentioned this procedure as a form of shared decision-making and experienced sufficient involvement in decision-making. Moreover, some parents stressed their preference not to be present at all consultations between professionals because of the burden on the family. A precondition for families feeling sufficiently involved in decision-making could lie in a conscious use of interprofessional consultation. This implies professionals using interprofessional consultation purposefully, embedded in families’ decision-making process and through transparency with families.

***Balancing roles of Families and Professionals.*** Making shared decisions with families with multiple and enduring problems faces professionals with dilemmas regarding their role in decision-making. On the one hand, SDM seeks to enable clients to exercise their autonomy and make decisions, leading to enhanced client alliance and engagement in treatment activities (Langer & Jensen-Doss, 2018). Likewise, professionals and families in our study stressed the importance of taking seriously families’ own goals, suggestions for support and prioritization of needed actions. On the other hand, especially with vulnerable clients - as youth and their families with multiple and enduring problems - professionals need to support decision-making (Bunn et al., 2018; Knutsson & Schön, 2020). In our study, professionals as well as families valued that professionals compensate for families’ limited capabilities when needed. Overall, professionals take an active role in supporting parents and youth in SDM, e.g., in raising awareness of problems and goals, providing overview to prioritize, and suggesting options of care. Thus, families and professionals are recommended to balance their mutual roles in decision-making in line with the needs and preferences of families throughout the SDM process. Moreover, these needs may change through the process because of moments of crisis and relapse, or, alternatively, growth in insight and coping skills (Nooteboom et al., 2020; Verwijmeren & Grootens, 2023). Also, a balance may be found between professional knowledge and families’ experiential knowledge. Therefore, SDM can be conceptualized as a continuum between professional-led decisions and decisions left entirely to families (Bjønness et al., 2020). Throughout the process, families and professionals regularly shift on this continuum as professionals need to take a more active lead, or when families develop in controlling their care process. Therefore, making shared decisions implies a continuous interplay between professionals and families, in which roles cannot be determined in advance but should be discussed regularly.

### Limitations and Strengths of this Study

The specific setting of Specialist Integrated care Teams in which we collected our data impacts the transferability of data (Daniel, 2019). We tried to mitigate this limitation by asking families and professionals broadly on their (previous) experiences in SDM, not limited to their experiences in SITs’ care. However, further research can give insight in whether our findings are feasible in other settings providing integrated care for families with complex needs.

We included participants with varying demographic characteristics considering age, educational level, and family structure, however the vast majority of parents was female and from Western backgrounds. As we know preferences and needs in SDM may vary across cultures, it is important to reach participants from different ethnic backgrounds in future studies (Qin et al., 2024). Although the interview transcripts were not returned to the participants for correction or comment, we did obtain feedback on the preliminary results from participants through learning sessions to increase the credibility of the study (Anney, 2014). Moreover, a parent representative participated in reflexive meetings on the study outcomes. Finally, the structured and theoretically grounded method in which the study was conducted, e.g., following the COREQ guidelines, using Makoul and Clayman’s well-researched SDM model and applying multiple analysis methods, further enhances the trustworthiness and credibility of this qualitative study (Daniel, 2019; Tong et al., 2007). Although providing a strong theoretical basis, applying Makoul and Clayman’s model may have limited what was asked and found. To mitigate this limitation, we asked participants broadly on their experiences in SDM (not specifically mentioning Makoul and Clayman’s essential elements) and analyzed both deductively on the known elements and inductively by open coding on other important elements.

## Conclusion

The integrated youth care context of families with multiple and enduring problems requires a specific approach in SDM. The known essential elements of SDM are realized with a broad view on the multiple family members, life domains, and professionals involved. Moreover, professionals are encouraged to consider a continuous and cyclical process of larger and smaller decisions and take time to build collaborative relationships with families and the care network. Throughout the SDM process families and professionals balance their mutual roles in decision-making in line with the needs and preferences of families. Further research is needed to explore the facilitators and barriers in implementing the SDM elements in practice from the perspectives of families and professionals.

## Electronic Supplementary Material

Below is the link to the electronic supplementary material.

Supplementary Material 1

Supplementary Material 2

## Data Availability

The datasets used and analyzed during the current study (transcript of the interviews) are not publicly available due to privacy related issues. Information on transcripts is available from the corresponding author on reasonable request. Interviewguides and topiclists are available in Appendix A.
