# Supplementary material for: Professionals’ and Families’ Perspectives on Essential Elements of Shared Decision-Making: A Qualitative Analysis on Families with Multiple and Enduring Problems in Integrated Youth Care
Source: Adm Policy Ment Health. 2025 Apr 30;52(4):640–52. doi: 10.1007/s10488-025-01443-0 (PMC12310899; doi:10.1007/s10488-025-01443-0)
Supplement: Supplementary file 1 — Supplementary Material 1 [file 10488_2025_1443_MOESM1_ESM.docx]

**Appendix B**

**Table B.1**

*Demographic Characteristics of Parents and Youth*

| Parents (*n*=18) |  | Youth (*n*=3) |  |
| --- | --- | --- | --- |
| *Gender* |  | *Gender* |  |
| Male | 4 (22,2%) | Male | 2 (66,7%) |
| Female | 14 (77,8%) | Female | 1 (33,3%) |
| Non-binary | 0 (0%) | Non-binary | 0 (0%) |
| *Age* |  | *Age* |  |
| 30 – 39 years | 2 (11,1%) | 15 years | 1 (33,3%) |
| 40 – 49 years | 8 (44,5%) | 16 years | 1 (33,3%) |
| 50 – 59 years | 6 (33,3%) | 17 years | 1 (33,3%) |
| Unknown | 2 (11,1%) | Unknown | 0 (0%) |
| *Cultural background* |  | *Cultural background* |  |
| Western | 17 (94,5%) | Western | 2 (66,7%) |
| Non Western | 1 (5,5%) | Non Western | 1 (33,3%) |
| *Highest educational level* |  | *Highest educational level* |  |
| Secondary Vocational Education | 9 (50,1%) | High School | 1 (33,3%) |
| University of Applied Sciences | 6 (33,3%) | Secondary Vocational Education | 1 (33,3%) |
| University | 1 (5,5%) | University of Applied Sciences | 1 (33,3%) |
| Unknown | 2 (11,1%) | Unknown | 0 (0%) |
| *Family structure* |  | *Family structure* |  |
| Two-parent household | 12 (66,7%) | Two-parent household | 1 (33,3%) |
| Single-parent household | 6 (33,3%) | Single-parent household | 2 (66,7%) |
| *Number of children*  One child | 2 (11,1%) | *Number of children*  One child | 0 (0%) |
| Two children | 9 (50,1%) | Two children | 0 (0%) |
| Three or more children | 7 (38,8%) | Three or more children | 3 (100%) |
| *SIT/region ^a^*  Beter Thuis/Haaglanden  In Verbinding/Midden-Holland  PAST/Midden-Holland  MAST/Alphen a/d Rijn  Katwijk | 4 (22,2%)  4 (22,2%)  2 (11,1%)  5 (27,8%)  3 (16,7%) | *SIT/region*  Beter Thuis/Haaglanden  In Verbinding/Midden-Holland  PAST/Midden-Holland  MAST/Alphen a/d Rijn  Katwijk | 1 (33,3%)  1 (33,3%)  1 (33,3%)  0 (0%)  0 (0%) |

*Note.*

^a^ From each SIT, an equivalent number of participating parents and youth were recruited, parents and youth were counted as one group (i.e. families).

**Table B.2**

*Demographic Characteristics of Professionals*

| Professionals (*n*=22) |  |
| --- | --- |
| *Gender*  Male  Female  Non-binary |  |
|  | 1 (5 %) |
|  | 21 (95%) |
|  | 0 (0%) |
| *Age*  20 – 29 years  30 – 39 years  40 – 49 years  50 – 59 years  60 – 69 years |  |
|  | 0 (0%)  8 (36%)  8 (36%)  5 (23%)  1 (5%) |
| *Work experience in years*  0 – 9 years  10 – 19 years  20 – 29 years  30 – 39 years  40 – 49 years |  |
|  | 5 (22%)  7 (32%)  7 (32%)  1 (5%)  2 (9%) |
| *Highest educational level*  Secondary Vocational Education  University of Applied Sciences  University |  |
|  | 1 (5%)  13 (59%)  8 (36%) |
| *Occupation*  Child and parent social worker  Psychologist/other therapist  Systemic therapist  Pediatric nurse  Child psychiatrist/youth physician |  |
|  | 13 (59%)  5 (22%)  1 (5%)  1 (5%)  2 (9%) |
| *Expertise*  Youth mental health  Youth and parenting support  Intellectual disabilities  Youth health service | 5 (23%) |
|  | 10 (45%) |
|  | 5 (23%) |
|  | 2 (9%) |
|  |  |
| *SIT/region*  Beter Thuis/Haaglanden  In Verbinding/Midden-Holland  PAST/Midden-Holland  MAST/Alphen a/d Rijn  Team in formation/Katwijk | 5 (23%)  3 (14%)  4 (18%)  4 (18%)  6 (27%) |
|  |  |
|  |  |
