# Supplementary material for: Professionals’ and Families’ Perspectives on Essential Elements of Shared Decision-Making: A Qualitative Analysis on Families with Multiple and Enduring Problems in Integrated Youth Care
Source: Adm Policy Ment Health. 2025 Apr 30;52(4):640–52. doi: 10.1007/s10488-025-01443-0 (PMC12310899; doi:10.1007/s10488-025-01443-0)
Supplement: Supplementary file 2 — Supplementary Material 2 [file 10488_2025_1443_MOESM2_ESM.docx]

**Appendix A: Topic Lists and Coding Framework of Semi-structured Interviews**

**Table A.1**

*Topic List Semi-structured Interviews Youth and Parents*

| Topic | Question |
| --- | --- |
| Family characteristics | Gender  Age  Family status and composition  Educational level  Type of problems (global description) |
| Shared decision-making of families and professionals | Introduction: During the care process, you as a family regularly make decisions together with the professionals, for example about the goals of care or about what support you want to receive as a family.  Can you/can you tell us something about how that shared decision-making works?  What is going well in this process and what is going not so well?  What do you think is important in making decisions together?  What role do you take in making decisions together and what do you prefer to leave to the professional? Does that role vary by decision or by period/phase in care?  How and to what extent do you experience control in the care process? What do you expect from the professional in taking control? |

**Table A.2**

*Topic List Semi-structured Interviews Professionals*

| Topic |  | Question |
| --- | --- | --- |
| Demographics and expertise | | Gender  Age  Educational level  Number of years of work experience  Care service and expertise |
| Shared decision-making | | How do you and the family make shared decisions, for example about the goals of support?  What do you think are important elements in shared decision-making with these families? |
|  | | What role do parents and/or youth take in shared decision-making? Does that role vary by decision or by period/phase in care?  What do you think that families need from professionals regarding control and coordination of care? How do you provide this? |

**Table A.3**

*Coding Framework Semi-structured Interviews*

| Code | Description code |
| --- | --- |
| Define or explain problem | Define and/or explain what the problem is that needs to be addressed, what goal needs to be achieved, or what decisions need to be made. |
| Present options | Present different options of care. |
| Discuss pros and cons (benefits/risks/costs) | Discuss the pros and cons of options: such as benefits, risks, costs, convenience. |
| Assess clients’ values or preferences | Evaluate the family's values, preferences, or experiential knowledge; Examining values and preferences of the family (young person and parents), including ideas, concerns and expectations (of the outcome). |
| Discuss client ability or self-efficacy | Discuss the family's ability or self-reliance to carry out a plan: Is the option feasible? (e.g. treatment/counselling/protocol, research, medication, behavioural change, referral). |
| Provide professional knowledge or recommendations | Provide professional knowledge, recommendations, or preferences of the professionals: in the context of the decision in question. |
| Check or clarify understanding | Throughout the process, check whether facts and perspectives are understood and, if necessary, clarify. |
| Make or explicitly defer decision | Make a decision or explicitly postpone it for a later date. |
| Arrange follow-up | Plan and execute follow-up to evaluate the effectiveness of decisions, make deferred decisions, or revise the treatment plan due to changed circumstances or preferences. |
| Elements of SDM (Open coding) | What is done (steps, elements or specific behaviors) to reach shared decision-making of family and professional(s) in care (on problems, goals, provided care) |

*Note.* Codes derived from Makoul & Claymans (2006) integrative model of SDM.
